# Differentiation of Cerebral Neoplasms with Vessel Size Imaging (VSI)

**DOI:** 10.1007/s00062-021-01129-8

**Published:** 2021-12-23

**Authors:** Asmaa Foda, Elias Kellner, Asanka Gunawardana, Xiang Gao, Martin Janz, Anna Kufner, Ahmed A. Khalil, Rohat Geran, Ralf Mekle, Jochen B. Fiebach, Ivana Galinovic

**Affiliations:** 1 International Graduate Program Medical Neurosciences, Charité – Universitätsmedizin Berlin, Hindenburgdamm 30, 12200 Berlin, Germany; 2 Center for Stroke Research Berlin, Charité – Universitätsmedizin Berlin, Berlin, Germany; 3 Department of Radiology, Medical Physics, University Medical Center Freiburg, Freiburg, Germany; 4 Institute of Biometry and Clinical Epidemiology, Charité – Universitätsmedizin Berlin, Berlin, Germany; 5 Berlin Institute of Health (BIH), Anna-Louisa-Karsch-Str. 2, 10178 Berlin, Germany; 6 Department of Hematology, Oncology and Cancer Immunology, Charité – Universitätsmedizin Berlin, Berlin, Germany; 7 Klinik und Hochschulambulanz für Neurologie, Charité – Universitätsmedizin Berlin, Berlin, Germany; 8 Berlin School of Mind and Brain, Humboldt Universität zu Berlin, Berlin, Germany; 9 Max Planck Institute for Human Cognitive and Brain Sciences, Berlin, Germany; 10 Department of Neurology with Experimental Neurology, Charité – Universitätsmedizin Berlin, Berlin, Germany

**Keywords:** Magnetic resonance imaging, Brain imaging, Brain tumors, Microvasculature, Differential diagnosis

## Abstract

**Purpose:**

Cerebral neoplasms of various histological origins may show comparable appearances on conventional Magnetic Resonance Imaging (MRI). Vessel size imaging (VSI) is an MRI technique that enables noninvasive assessment of microvasculature by providing quantitative estimates of microvessel size and density. In this study, we evaluated the potential of VSI to differentiate between brain tumor types based on their microvascular morphology.

**Methods:**

Using a clinical 3T MRI scanner, VSI was performed on 25 patients with cerebral neoplasms, 10 with glioblastoma multiforme (GBM), 8 with primary CNS lymphoma (PCNSL) and 7 with cerebral lung cancer metastasis (MLC). Following the postprocessing of VSI maps, mean vessel diameter (vessel size index, vsi) and microvessel density (Q) were compared across tumors, peritumoral areas, and healthy tissues.

**Results:**

The MLC tumors have larger and less dense microvasculature compared to PCNSLs in terms of vsi and Q (*p* = 0.0004 and *p* < 0.0001, respectively). GBM tumors have higher yet non-significantly different vsi values than PCNSLs (*p* = 0.065) and non-significant differences in Q. No statistically significant differences in vsi or Q were present between GBMs and MLCs. GBM tumor volume was positively correlated with vsi (r = 0.502, *p* = 0.0017) and negatively correlated with Q (r = −0.531, *p* = 0.0007).

**Conclusion:**

Conventional MRI parameters are helpful in differentiating between PCNSLs, GBMs, and MLCs. Additionally incorporating VSI parameters into the diagnostic protocol could help in further differentiating between PCNSLs and metastases and potentially between PCNSLs and GBMs. Future studies in larger patient cohorts are required to establish diagnostic cut-off values for VSI.

**Supplementary Information:**

The online version of this article (10.1007/s00062-021-01129-8) contains supplementary material, which is available to authorized users.

## Introduction

Vessel size imaging (VSI) is a magnetic resonance imaging (MRI) technique that allows in vivo examination of cerebral microvascular morphology by providing quantitative estimates of mean vessel diameter and density within a given voxel [1–3]. The MR-VSI is based on exploiting the ratio of relaxation rate changes (Δ R*_2_/ΔR_2_) measured by gradient echo (GE) and spin echo (SE) pulse sequences in vascular networks during the passage of an intravascular contrast agent [1, 4]. The T2*-weighted and T2-weighted images generated from GE and SE during dynamic susceptibility contrast (DSC) perfusion imaging are sensitive to large vessels and microvasculature < 10 μm in diameter, respectively [4]. Thus, VSI combines two different contrasts that are sensitive to a specific range of vessel sizes each.

Troprès et al. [5] showed that VSI enables a clear differentiation of intratumoral, peritumoral, and healthy contralateral tissue in an animal model of glioma. Further work by Kiselev et al. [3] demonstrated the feasibility of using this technique in studying human tumors by generating quantitative measures of the average microvessel diameter (vessel size index, vsi) using the apparent diffusion coefficient (ADC) obtained from an additional diffusion-weighted MRI scan, relative cerebral blood volume (rCBV), and mean microvessel density (Q). Several animal studies thereafter employed MR-VSI in assessing the vascular development of various tumors, including brain tumors [2, 5, 6], subcutaneously induced solid tumors [7–9] as well as monitoring vascular remodeling during therapeutic interventions [7, 9, 10]. Moreover, several clinical studies demonstrated the reliability and feasibility of this technique in providing quantitative estimates of the pathological changes in the microvascular networks of brain tumors [3, 11] and ischemic stroke [12, 13].

Several validation studies, both preclinical and clinical, depicted a high correlation between the microvascular measures obtained by VSI and those obtained by histopathology [14–17]. The VSI also proved useful in differentiating glioma subtypes and predicting tumor grades [15, 17, 18]. In addition, a technique similar to VSI yet with more advanced postprocessing has been used in isolated studies of monitoring vascular responses in tumor patients receiving antiangiogenic treatment with promising results [19]; however, the potential utility of VSI in differentiating between cerebral neoplasms of different etiologies based on the quantitative properties of their microvasculature has not yet been investigated.

Different tumor types can elicit entirely different patterns of vascularization [20, 21]. We hypothesized that microvessel diameter and microvessel density values assessed by VSI will be different enough across different types of malignant cerebral neoplasms to assist in their differential diagnosis. If successful, this method could provide new MRI-based biomarkers for the preoperative differentiation of various intracerebral neoplasms.

## Material and Methods

### Study Design

This project is part of an ongoing proof of concept study (MINC: vessel size imaging as a novel MRI method for assessing **mi**crovasculature in **n**eurological **c**onditions) carried out at the Neuroradiology Group of the Center for Stroke Research Berlin (CSB). The local ethics committee of the Charité Universitätsmedizin Berlin (Campus Benjamin Franklin) approved the study (#EA4/131/19).

### Patients

Our cohort consisted of 25 patients (11 females, 14 males) with a median age of 71 years (range 40–87 years). Patients of this pilot study were enrolled over a period of 10 months (between November 2019 and July 2020). Included were patients who presented with pathological intracerebral masses and from whom written consent was obtained. Excluded were patients with poor image quality introduced due to motion artifacts preventing reliable image analysis. Our cohort consisted of 10 patients with a WHO grade IV glioblastoma multiforme (GBM), 8 patients with primary central nervous system lymphomas (PCNSL) and 7 patients with metastases of lung cancer (MLC). The diagnosis was confirmed by histopathology in all cases. All patients gave written informed consent for the experimental protocol. Of the patients, 19 were scanned prior to any treatment whereas 6 patients had received either chemotherapy or radiotherapy 1 year to a few days prior to the MRI scan. Patients who received treatment within the last 6 months before scanning were later excluded from a subanalysis of our study (*n* = 5).

### Magnetic Resonance Imaging (MRI) Acquisition

All measurements were performed using a clinical 3T Prismafit MRI scanner (Siemens Healthineers; Erlangen, Germany) with a 64-channel head coil. The VSI was performed using repeated single-shot gradient echo (GE) spin echo (SE) echo planar readouts during the intravascular passage of a paramagnetic contrast agent bolus (10 ml Gadovist® 1.0 mmol/ml, Bayer AG, Leverkusen, Germany) at a flow rate of 5 ml/s was followed by a bolus of 30 ml isotonic saline flush. Two versions of the VSI sequence were used (standard sequence with TR = 2000 ms, TE = 18 ms and 84 ms for GE and SE, respectively, flip angle 90º and an alternative protocol with TR = 2100 ms, TE = 18 ms and 92 ms, respectively) because technical problems occasionally arose (in 7 patients) necessitating the replacement of the standard protocol. The voxel resolution was 1.8 × 1.8 × 5.5 mm^3^ with a total of 16 slices and full supratentorial brain coverage for both protocols. Parameters of the VSI sequence are adapted from [15] using single-band excitation, which has a comparable signal-to-noise ratio to the multiband method [22].

In total, 50 dynamic volumes were acquired during contrast administration. Additional acquired sequences in the examination were: diffusion-weighted imaging (DWI), fluid-attenuated inversion recovery (FLAIR), and a contrast-enhanced 3D T1-weighted high-resolution magnetization-prepared rapid acquisition gradient echo (MPRAGE) sequence.

### Data Processing

The dynamic susceptibility measurements were processed similar to [15] with the following procedure: first, the change in relaxation rates for both GE and SE were calculated using the proportionality to log(S_(t)_/S_0_), where S_0_ denotes the baseline signal, calculated as average over the first 5 timepoints of the time series. Then, a T1 leakage correction was performed [11]. From this, the first bolus passage was extracted with the procedure described in [23]. According to [3], the mean vessel diameter d relates $\Delta R_{2}^{*}$ and Δ*R*_2_ via

1 $$\Updelta {R_{2}^{\mathrm{*}}}^{2/3} = \left(\frac{d}{1.73.\left(\mathrm{rCBV}.\mathrm{ADC}\right)^{1/2}} \right)^{2/3} \Updelta R_{2}$$

2 $$Q\equiv \frac{\Delta R_{2}}{\Delta R_{2}^{*\:2/3}}$$

$\Delta R_{2}^{*}$ and Δ*R*_2_ represent the changes in the relaxation rates for GE and SE acquisitions due to the administration of contrast agent, respectively, *d* is the mean vessel diameter calculated in μm, *rCBV* is the cerebral blood volume fraction normalized to a global average of 3.2% and *ADC* is the apparent diffusion coefficient. Exploiting Eq 1, a fit to the measured datapoints of $\Delta {R_{2}^*}^{2/3}$ and Δ*R*_2_ was performed with a linear Deming regression, which correctly accounts for errors on both axes. From the fitted regression coefficient, the mean vessel diameter *d* was calculated. From the same linear fit, the mean vessel density *Q* as given in Eq 2 and defined in [24] was calculated.

Only timepoints during the bolus passage were used for curve fitting by setting a threshold to the measured time series, $\Delta R_{2}^{*}$ and Δ*R*_2_. Furthermore, a threshold of 200 μm was applied to mean vessel diameter maps to exclude (null) voxels, which were beyond the limits of the mathematical model.

Perfusion maps of relative cerebral blood volume (rCBV) and relative cerebral blood flow (rCBF) were calculated using the VEOcore Software (VEObrain GmbH, Freiburg, Germany) [24] based on the gradient echo images from the VSI scans. The rCBV was calculated as the integral over the Δ*R*_2_ after leakage correction and first bolus extraction and rCBF was determined using a deconcolution with an arterial input function. All image processing and annotation were performed using MATLAB-based scripts imbedded into a local installation of the NORA imaging platform (www.nora-imaging.org).

### Delineation of Regions of Interest (ROIs)

Blinded to vsi and Q maps, regions of interest were manually delineated by A. F. (master’s student) and validated by I.G. (neuroradiologist with 10 years experience) on the high-resolution contrast-enhanced T1-weighted images, or on T2-weighted FLAIR images in the case of a non-enhancing tumor. Only one PCNSL patient had non-enhancing tumor lesions with discrete areas of T2-hyperintensity, representing a clear progress of the tumor based on a comparison with a recent imaging time point.

**Fig. 1:**
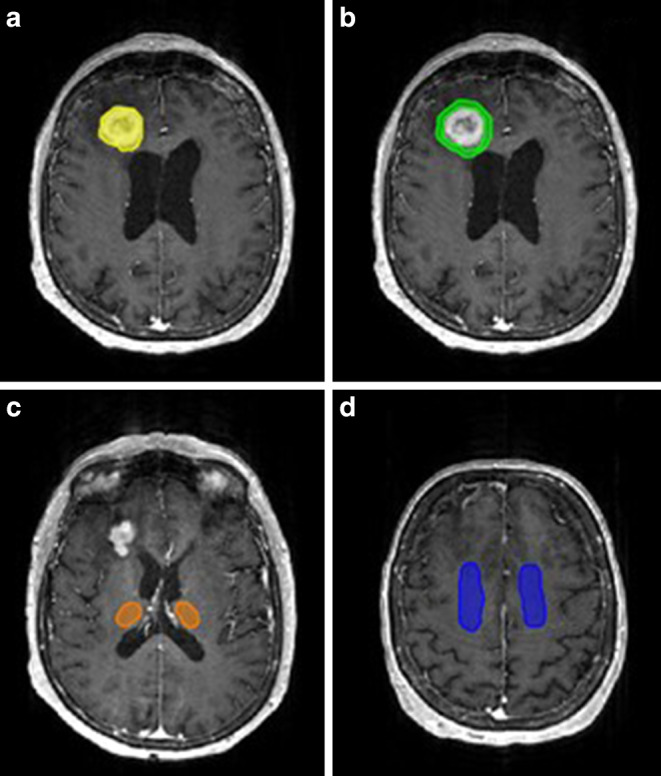
Manual selection of the regions of interest (ROIs) on postcontrast T1-weighted images. **a** tumor (*yellow*), **b** peritumoral area (*green*), **c** healthy gray matter from both thalami (*orange*), and **d** healthy white matter from the centrum semiovale (*blue*)

For each patient, at least 4 ROIs were obtained for evaluation (Fig. 1):

1. The whole tumor, obtained by delineating the contrast-enhancing segment of the tumor and taking care to exclude necrotic areas (if present) (Fig. 1a),
2. Peritumoral areas, obtained by expanding the original tumor ROI by 4 voxels along each axis, then subtracting the original tumor ROI from the dilated one (Fig. 1b),
3. Healthy gray matter tissue (GM) collected from both thalami (Fig. 1c), and
4. Healthy white matter (WM) obtained mainly from the centrum semiovale (Fig. 1d).

In cases where multiple neoplastic lesions existed, each of them was separately delineated and treated as a distinct tumor. Only tumor lesions ≥ 0.1 ml in volume were included in the analysis, as smaller tumors might deliver imprecise results due to noise and partial volume effects. The VSI and perfusion maps were registered to the T1-weighted images. All defined ROIs were then superimposed on VSI and perfusion maps for the quantitative estimation of median vsi in μm, median Q in s^−1/3^, relative cerebral blood volume (rCBV) and cerebral blood flow (rCBF) (percentage relative to the contralateral hemisphere).

### Statistical Analysis

The study involved 25 patients in total with 3 different types of brain tumors and different numbers of lesions per patient. Here, a patient is considered as a single cluster and the lesions per patient as dependent replicates. Since the sample sizes are rather small, inferring any distributional assumptions of the data (e.g., normality and/or variance homoscedasticity) would be questionable. Therefore, we analyzed the data using a purely nonparametric approach for dependent replicates proposed by [36].

Note that although the test statistics derived in [36] are applicable for two independent samples with replicated observations, we have modified its test procedure to take three independent groups of this study into account. Furthermore, we consider the weighted scheme described in [36] as the size of each cluster is rather different. Comparisons between healthy tissues of GM and WM were performed using Welch’s t‑test or Mann-Whitney test, depending on the normality of data. The level of significance was set to 5% and multiple comparisons were adjusted for using Bonferroni correction. The statistical analyses were performed using R version 4.0.3 (https://www.r-project.org/).

## Results

### VSI Maps of the Different Brain Tumor Types

**Fig. 2:**
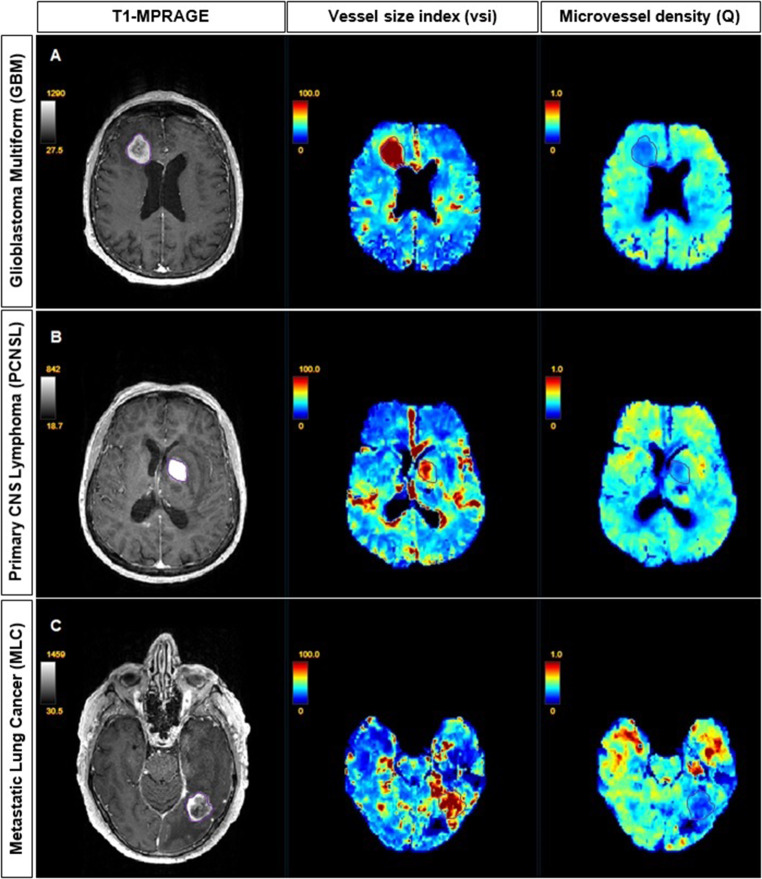
VSI maps shown using the NORA medical imaging platform in patients with **a** glioblastoma multiforme, **b** primary CNS lymphoma, **c** metastatic lung cancer. *Left:* post-contrast T1-weighted images showing enhancement of tumor regions. *Middle:* corresponding mean vessel diameter (vsi) maps. *Right*: corresponding microvessel density (Q) maps. Tumor regions show elevated mean vessel diameters with decreased microvessel densities compared to healthy tissues

Fig. 2 depicts examples of the resultant mean vessel diameter (vsi) and microvessel density (Q) maps of patients with different tumor etiologies.

### Mean Vessel Diameter (vsi) and Microvessel Density (Q) Values.

Table 1 summarizes the median and interquartile range (IQR) values of vsi and Q in tumors, peritumoral areas, and healthy tissues. We compared the microvessel diameter and microvessel density values across three different tumor types: glioblastoma multiforme (GBM; N_cases_ = 10, N_lesions_ = 37), primary CNS lymphomas (PCNSL; N_cases_ = 8, N_lesions_ = 30), and metastatic lung cancer (MLC; N_cases_ = 7, N_lesions_ = 31).

**Table 1:** Summary of median and interquartile range (IQR) values of vsi and Q in tumors, peritumoral areas, and healthy tissues

Diagnosis	GBM	PCNSL	MLC	Healthy GM	Healthy WM
***Vessel size index (vsi)***					
**Median (IQR) in μm**					
*Tumor*	82.26 (47.39–92.87)	49.74 (40.54–68.02)	82.34 (55.2–112.2)	30.04 (25.14–35.76)	24.48 (19.78–40.02)
*Peritumoral area*	50.32 (36.67–63.17)	47.53 (36.67–54.28)	58.74 (44.88–70.30)		
***Microvessel density (Q)***					
**Median (IQR) in s**^**−1/3**^					
*Tumor*	0.286 (0.246–0.396)	0.326 (0.278–0.399)	0.266 (0.219–0.313)	0.449 (0.404–0.506)	0.411 (0.37–0.449)
*Peritumoral area*	0.348 (0.31–0.446)	0.35 (0.307–0.387)	0.315 (0.288–0.351)		

*GBM* glioblastoma multiforme, *PCNSL* primary CNS lymphoma, *MLC* metastatic lung cancer, *GM* gray matter, *WM* white matter

**Fig. 3:**
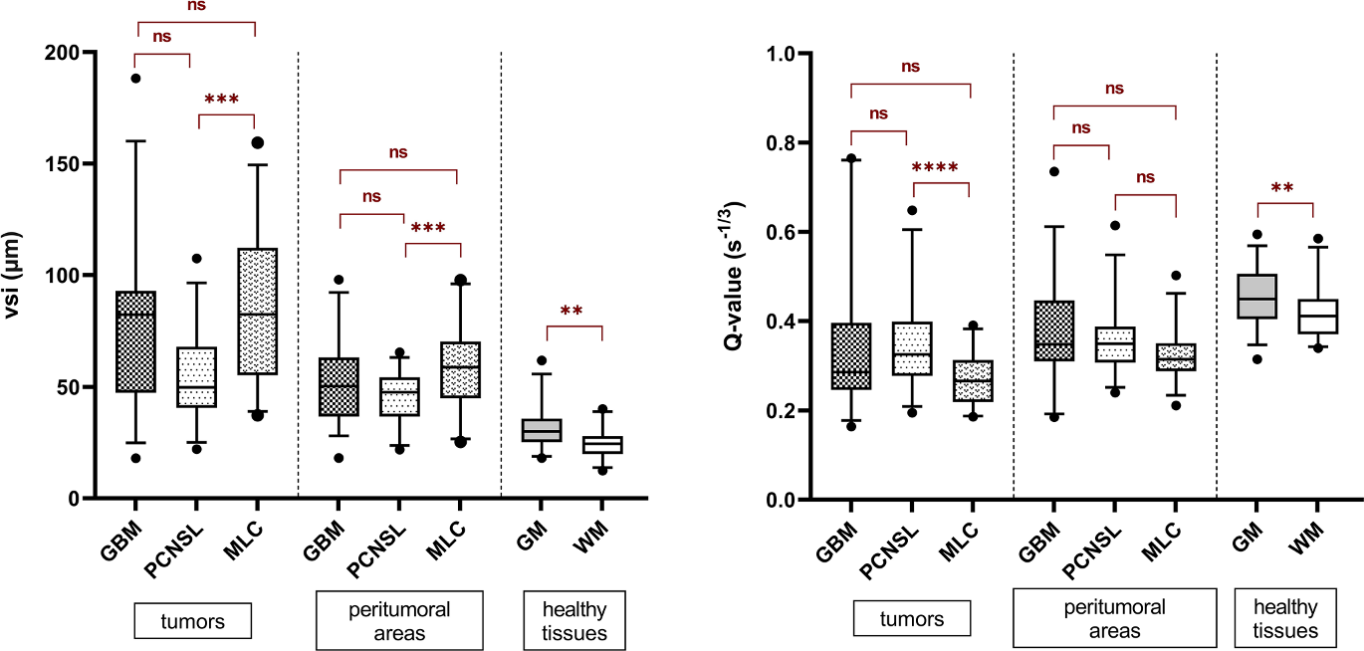
Boxplot representation of vessel size index (vsi) (**a**) and microvessel density (Q) (**b**) in different tumor types, their surrounding areas, and healthy gray and white matters. Whiskers indicate 5–95 percentiles. *GBM* glioblastoma multiforme, *PCNSL* primary CNS lymphoma, *MLC* metastatic lung cancer, *GM* gray matter, *WM* white matter

The MLC showed significantly larger microvessel size diameters compared to PCNSL, (*p* = 0.0004). GBM had larger vsi values than PCNSL, however, this difference did not reach statistical significance (*p* = 0.065). No significant difference in vsi was found between MLC and GBM tumors (*p* = 0.971). Regarding the measure of microvessel density (Q), only PCNSL tumors showed denser microvessels compared to MLCs (*p* < 0.0001) but no significant difference was found between GBM and PCNSL tumors (*p* = 0.942) or between GBM and MLC tumors (*p* = 0.119). In peritumoral areas the only significant difference was in vessel sizes between MLC and PCNSL (*p* = 0.0005) where the former showed larger vessel sizes than the latter. Healthy GM tissues showed both significantly larger and denser microvasculature than healthy WM (*p* = 0.0015 and *p* = 0.022, respectively). Boxplot representations of the distribution of vsi and Q values across tissue types are shown in Fig. 3. All tumor lesions, regardless of the tumor type, showed significantly larger microvessel sizes and lower microvessel densities in comparison to healthy tissues. Generally, vsi was highest and Q lowest within the tumor lesions, followed by peritumoral areas and lastly healthy tissues of gray and white matters (*p* < 0.005 for all). Results remained unchanged in a subanalysis of only those tumor lesions that were either naïve to treatment or were not subject to any treatment for the past 6 months (30/37 GBM lesions, 19/30 PCNSL lesions, and 28/31 MLC lesions).

### Perfusion and Diffusion Parameters.

Cerebral blood volume (CBV) and cerebral blood flow (CBF) expressed in % relative to the contralateral hemisphere, as well as apparent diffusion coefficient (ADC) values were also calculated and compared between tumors and healthy tissues (see Table 2).

**Table 2:** Summary of median and interquartile range (IQR) values of CBV, CBF, and ADC in tumors, peritumoral areas, and healthy tissues

	GBM	PCNSL	MLC	Healthy GM	Healthy WM
***Cerebral blood volume (CBV)***					
**Median (IQR) in %**					
*Tumor*	138.1 (86.74–215.9)	89.78 (64.38–121.9)	105.8 (70.9–133.9)	120 (108.1–143.2)	47.65 (39.11–58.11)
*Peritumoral area*	121.1 (88.28–153.6)	88.94 (59.01–115.5)	79.77 (60.02–104.9)		
***Cerebral blood flow (CBF)***					
**Median (IQR) in %**					
*Tumor*	139.7 (94.22–186.5)	106.6 (57.83–148.4)	88.01 (64.25–123.1)	130.2 (114.5–138.8)	41.09 (36.84–50.04)
*Peritumoral area*	126.5 (98.27–142.7)	93.68 (55.72–137.4)	68.34 (59.73–93.7)		
***Apparent diffusion coefficient (ADC)***					
**Median (IQR) in μm**^**2**^**/ms**					
*Tumor*	1.013 (0.817–1.21)	0.948 (0.839–1.172)	1.189 (1.046–1.371)	0.745 (0.709–0.79)	0.782 (0.746–0.809)
*Peritumoral area*	1.023 (0.821–1.149)	0.982 (0.869–1.114)	1.139 (1.046–1.333)		

*GBM* Glioblastoma multiforme, *PCNSL* Primary CNS Lymphoma, *MLC* Metastatic Lung Cancer, *GM* gray matter, *WM* white matter

The GBM tumors and the peritumoral areas showed significantly higher CBV than PCNSLs (*p* = 0.0001 and *p* = 0.0003, respectively). GBM tumors also had significantly higher CBF values compared to PCNSL (*p* = 0.0488) whereas the peritumoral areas had higher yet not significantly different CBF values (*p* = 0.08). Moreover, GBM tumors and their peritumoral areas showed significantly higher CBV (*p* = 0.035 and *p* = 0.037) and CBF values (*p* = 0.023 and *p* = 0.030) compared to the corresponding areas of MLC. No significant difference in either perfusion parameter was found between MLC and PCNSLs.

Regarding diffusivity, ADC values were significantly higher in MLC tumors than in both GBM (*p* = 0.001) and PCNSL (*p* = 0.005). Similarly, the peritumoral areas had significantly higher ADC values when compared to either GBM (*p* = 0.001) or PCNSL (*p* = 0.0001). ADC values were found non-significant between tumors and peritumoral areas of GBM vs. PCNSL (*p* > 0.05).

### Correlation of VSI Parameters with Tumor Volumes.

Spearman correlation coefficient was computed to assess the relationship between tumor size and the characteristics of its supplying microvasculature. Out of the 98 tumor lesions in our sample 79 lesions were included in the analysis (37 GBM, 30 PCNSL and 12 MLC) with 19 lesions excluded due to necrotic cores with viable tissue only at the edges since their ROIs would not be representative of the actual tumor volume.

**Fig. 4:**
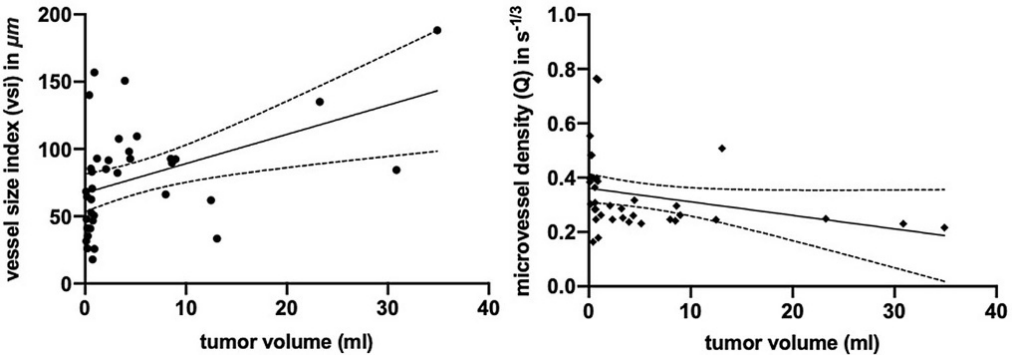
Scatterplot representation of the correlation analysis between tumor volumes and VSI parameters within glioblastoma multiforme (GBM) tumors. **a** Vessel size index (vsi) shows a relatively strong positive linear relationship with tumor volume (*r* = 0.502, *n* = 37, *p* = 0.0017). **b** Microvessel density (Q) shows a relatively strong negative correlation with tumor volume (*r* = −0.531, *n* = 37, *p* = 0.0007). Error bars indicate 95% CI. *Note: *various types of noise (image acquisition, co-registration, partial volume effect, etc.) may contribute to a certain variability in the final datapoints in the figure. This is particularly true for lesions with very small volume

Separated into groups based on tumor type, there was a significant positive correlation between GBM tumor volume and microvessel sizes (r = 0.502, *p* = 0.0018) and a significant negative correlation with microvessel density (Q) (r = −0.531, *p* = 0.0007). This indicates that increases in tumor size are correlated with larger yet less dense microvasculature (Fig. 4). No significant correlation was found for PCNSLs and MLCs.

Equally, no significant correlations were found between tumor volumes and any of the perfusion or diffusion parameters (Supplementary Table 1).

## Discussion

Accurate differential diagnosis of malignant cerebral neoplasms using conventional MRI techniques remains challenging in everyday practice. In the brain, glioblastoma multiforme (GBMs), primary CNS lymphomas (PCNSLs), and metastases belong to the most frequently encountered malignancies. All of them also show comparable appearances on conventional MRI sequences as solitary or multifocal, strongly enhancing, non-calcified, soft tissue masses surrounded by edema. The purpose of this study was to evaluate the usefulness of an MRI technique, vessel size imaging (VSI), in the preoperative differentiation of these tumors. In our study, we focused on comparing tumors and their surrounding areas, since both compartments provide important pathophysiological information necessary for tumor identification.

Our results showed that tumor microvasculature of MLC had significantly larger microvessel diameters compared to PCNSLs, whereas microvessel size did not significantly differ between MLC tumors and GBMs nor between PCNSLs and GBMs. A histopathological study by Gi et al. demonstrated that metastatic brain tumors, similar to high-grade gliomas, are characterized by a large, thick, dilated glomeruloid microvasculature compared to normal brain tissue or malignant lymphomas [26], which could explain the non-significant difference in the mean vessel diameters between GBMs and MLCs. The lack of statistical significance in the comparison of microvessel size and density between GMBs and PCNSLs could be attributed to the small sample size but partially also to the fact that GBMs seem to alter their microvasculature as they evolve, with smaller tumors presenting with smaller vessels more similar to PCNSLs (which are less vascular and exhibit a characteristic angiocentric pattern in which tumor cells accumulate densely within and around blood vessels [27]). All peritumoral areas showed microvessel diameters that were significantly smaller than the corresponding tumors yet significantly larger than normal brain tissues. A similar, yet inverse, gradient was revealed for the parameter Q, both possibly indicating a gradual metamorphosis of one tissue type to the other.

Histologically, and in contrast to GBMs and metastatic tumors, PCNSLs lack abundant neovascularization and tumor angiogenesis which matches our results of denser microvascular networks with smaller vessel caliber and lower rCBV. Our results are in line with several perfusion studies [28–30], which found that PCNSLs have lower perfusion than high-grade gliomas attributed to their different vascularity. VSI can therefore serve as a complementary method to aid the precise differentiation between MLC and PCNSL entities where perfusion parameters fail. The tumors and peritumoral areas of GBMs showed significantly higher perfusion (rCBV and rCBF) compared to those of MLCs which could be attributed to the highly infiltrative nature of glioblastomas as opposed to metastases, which are surrounded by pure vasogenic edema that leads to a local compression of microcirculation [31]. Indeed, several studies showed that peritumoral rCBV has a high diagnostic accuracy in discriminating between GBMs and metastases, where the former showed significantly higher values than the latter [30, 32, 33].

In contrast to standard perfusion, in GBMs VSI detected a correlation between tumor volume and the values of vsi and Q. Hence, smaller GBM tumors could have vessel sizes that are comparable to those of PCNLS but start resembling MLCs with more enlarged vessels as they gain size during their malignant progression. Comparing differently sized GBM tumors to PCNSLs and MLCs might be of interest to follow up in future studies with larger patient cohorts.

Estimation of vsi and Q in GBMs using the same VSI sequence has been previously reported by Kellner et al. [15], where they showed median (IQR) values of 74.5 μm (59.25–81.25) and 0.305 s^−1/3^ (0.29–0.387), respectively. These results were not significantly different from ours (*p* = 0.397 and *p* = 0.286, respectively). This consistency could further substantiate the reliability of VSI as a method to estimate microvascular characteristics. Indeed, several clinical studies showed that vessel sizes of high-grade gliomas obtained by VSI significantly correlated with those estimated by histopathology [16, 25]; however, it is important to note that these estimated vsi values do not necessarily accurately reflect actual microvascular anatomy. This is due to a known inherent systematic error of the method which leads to overestimation of normal vessel sizes and underestimation of grossly enlarged vessels [2, 15].

Classical MRI techniques, such as DWI and DSC perfusion-weighted imaging are already widely applied in diagnostic imaging of intracranial tumors, and several previous studies have shown differences in perfusion and diffusion values between GBMs, PCNSLs, and/or metastases [28, 30, 34, 35]. Standard DSC utilizes GE contrast only whereas VSI utilizes both dynamic GE and SE. As previously mentioned, GE is sensitive to vessels of all sizes while SE is weighted toward microvascular structures < 10 μm in diameter [4]. Therefore, while VSI as a technique incorporates conventional DSC imaging, it is also able to provide additional insight into the properties of microvasculature, which are often concealed in pure GE measurements, predominated by large vessels. Moreover, as VSI is designed to deliver quantitative parameters, it also enables intersubject comparison. The VSI sequence implemented on our clinical MRI scanner possesses identical spatial resolution in comparable scan time and contrast agent dosage to standard DSC MRI and can therefore be used interchangeably and feasibly as part of a routine diagnostic work-up for patients with suspected intracranial neoplasms. Considering that our department is primarily devoted to cerebrovascular diseases, a general limitation of our study is the small sample size of only 25 randomly selected brain tumor patients and the heterogeneous composition of the cohort with tumors of different treatment status. Another limitation is the inclusion of a patient with few non-enhancing lesions as well as the assessment of several lesions in a single case as distinct tumors; however, this was compensated by using a novel yet scientifically validated statistical method.

Current developments in the field involve a technique called vessel architectural imaging (VAI), which can extract, through advanced post-processing, even more information about the microvascular structures (such as the predominance of venules or arterioles in a voxel) using the same sequence as VSI [19, 22]. Building upon VAI, there is an opportunity to gain even deeper insights into the complexity and heterogeneity of vascular changes in cerebral neoplasms. In conclusion, this is the first study to evaluate the potential of VSI in differentiating between different etiologies of cerebral neoplasms and our findings support the utilization of VSI as an add-on to conventional MRI to facilitate tumor differentiation.

## Supplementary Information

**Results of the correlation analysis between the different tumor volumes and their vessel size index (vsi), perfusion, and diffusion parameters. Supplementary Table 1.** Summary of the Spearman correlation coefficient analysis results between tumor volumes vs vessel size index, perfusion, and diffusion parameters. *GBM* glioblastoma multiforme, *PCNSL* primary CNS lymphoma, *MLC* metastatic lung cancer, *vsi* mean vessel diameter, *q* microvessel density, *CBV* cerebral blood volume, *CBF* cerebral blood flow, *ADC* apparent diffusion coefficient, *rs* Spearman’s rank correlation coefficient; **p < 0.01, ***p < 0.001.
