# Supplementary material for: Differentiation of Cerebral Neoplasms with Vessel Size Imaging (VSI)
Source: Clin Neuroradiol. 2021 Dec 23;32(1):239–48. doi: 10.1007/s00062-021-01129-8 (PMC8894153; doi:10.1007/s00062-021-01129-8)
Supplement: Supplementary file 1 — Results of the correlation analysis between the different tumor volumes and their vessel size index (vsi), perfusion, and diffusion parameters. Supplementary Table 1. Summary of the Spearman correlation coefficient analysis results between tumor volumes vs vessel size index, perfusion, and diffusion parameters. GBM glioblastoma multiforme, PCNSL primary CNS lymphoma, MLC metastatic lung cancer, vsi mean vessel diameter, q microvessel density, CBV cerebral blood volume, CBF cerebral blood flow, ADC apparent diffusion coefficient, rs Spearman’s rank correlation coefficient; **p < 0.01, ***p < 0.001. [file 62_2021_1129_MOESM1_ESM.docx]

| **Group** | **Lesion volume**  **vs** | **r_s_** | ***p*-value** |
| --- | --- | --- | --- |
| **GBM** | **vsi** | 0.5021337 | 0.001761** |
|  | **Q** | -0.5315465 | 0.0007106*** |
|  | **CBV** | 0.173542 | 0.3031 |
|  | **CBF** | 0.1095306 | 0.5173 |
|  | **ADC** | -0.09685833 | 0.5685 |
| **PCNSL** | **vsi** | 0.1368187 | 0.4693 |
|  | **Q** | -0.08031146 | 0.6721 |
|  | **CBV** | 0.2111235 | 0.2616 |
|  | **CBF** | 0.3259177 | 0.07925 |
|  | **ADC** | -0.01490711 | 0.9377 |
| **MLC** | **vsi** | 0.2797203 | 0.3787 |
|  | **Q** | -0.4965035 | 0.1041 |
|  | **CBV** | -0.01398601 | 0.9737 |
|  | **CBF** | -0.02097902 | 0.9562 |
|  | **ADC** | 0.0979021 | 0.7663 |

**Supplementary Table 1.** Summary of the Spearman correlation coefficient analysis results between tumor volumes vs VSI, perfusion, and diffusion parameters. GBM: Glioblastoma multiforme; PCNSL: Primary CNS Lymphoma; MLC: Metastatic Lung Cancer; vsi: vessel size index; Q: microvessel density; CBV: cerebral blood volume; CBF: cerebral blood flow; ADC: apparent diffusion coefficient; r_s_: Spearman's rank correlation coefficient; ***p*<0.01, ****p*<0.001.
